# Growth, Physiology and Yield of Traditional Cowpea Varieties Under Salt Stress Using Exogenous Magnesium

**DOI:** 10.3390/plants14223524

**Published:** 2025-11-19

**Authors:** Antonio Sávio dos Santos, Miguel Ferreira Neto, Hayanne Ywricka de Araújo Melo, Ricardo André Rodrigues Filho, Francisca das Chagas de Oliveira, Joyce Fernandes de Medeiros, Clara Araújo da Silva, Paula Cristina de Morais Rosario, José Francismar de Medeiros, Nildo da Silva Dias, Tayd Dayvison Custódio Peixoto, Josinaldo Lopes Araújo, Alberto Soares de Melo, Alex Álvares da Silva, Francisco Vanies da Silva Sá

**Affiliations:** 1 Department of Agronomic and Forest Sciences, Universidade Federal Rural do Semi-Árido, Mossoró 59625-900, RN, Brazil; antonio.santos63350@alunos.ufersa.edu.br (A.S.d.S.); miguel@ufersa.edu.br (M.F.N.); hayanne.melo@alunos.ufersa.edu.br (H.Y.d.A.M.); ricardo.filho45389@alunos.ufersa.edu.br (R.A.R.F.); francisca.oliveira58483@alunos.ufersa.edu.br (F.d.C.d.O.); joyce.medeiros55308@alunos.ufersa.edu.br (J.F.d.M.); clara.silva55106@alunos.ufersa.edu.br (C.A.d.S.); paula.rosario@alunos.ufersa.edu.br (P.C.d.M.R.); jfmedeir@ufersa.edu.br (J.F.d.M.); nildo@ufersa.edu.br (N.d.S.D.); 2 Center of Agrarian and Biological Sciences, Universidade Estadual Vale do Acaraú, São Benedito 62370-000, CE, Brazil; tayd_custodio@uvanet.br; 3 Academic Unit of Agricultural Engineering, Universidade Federal de Campina Grande, Campina Grande 58429-900, PB, Brazil; josinaldo.lopes@professor.ufcg.edu.br; 4 Department of Biological Sciences, Universidade Estadual da Paraíba, Campina Grande 58429-900, PB, Brazil; alberto.melo@servidor.uepb.edu.br; 5 Department of Agrarian and Exact, Universidade Estadual da Paraíba, Catolé do Rocha 58884-000, PB, Brazil; alex.alvares@visitante.uepb.edu.br

**Keywords:** *Vigna unguiculata* (L.) Walp, biosaline agriculture, plant nutrition, osmotic stress

## Abstract

Salinization is one of the main environmental challenges affecting agriculture in semi-arid regions. We evaluated the feasibility of foliar magnesium and its effects at different doses on the acclimation of cowpea varieties under salt stress. The experiment occurred in a greenhouse using a randomized block design in a 2 × 3 × 4 factorial scheme, with five replicates. Two cowpea varieties—‘Pingo de Ouro’ and ‘Costela de Vaca’—were subjected to three salinity levels in irrigation water (0.54, 3.50, and 5.00 dS m^−1^) and four foliar magnesium (Mg) doses (0, 1, 2, and 3 mL L^−1^). Under 3.50 dS m^−1^ salinity, the 1 mL L^−1^ dose resulted in the highest yield per plant (18.29 g). CO_2_ assimilation was highest with 2 mL L^−1^ Mg at 3.50 dS m^−1^ for ‘Costela de Vaca’, and with 1 mL L^−1^ Mg at 5.00 dS m^−1^ for ‘Pingo de Ouro’. The ‘Pingo de Ouro’ variety was more tolerant to ‘Costela de Vaca’. Foliar Mg fertilization proved to be a promising strategy to mitigate the effects of salt stress in cowpea, especially for ‘Pingo de Ouro’. Magnesium effectively reduces salt stress, but its effect varies by plant variety and irrigation salinity, necessitating customized dose adjustments.

## 1. Introduction

Cowpea (*Vigna unguiculata* (L.) Walp) is a crop of significant socioeconomic and nutritional importance in several regions of the world, particularly in tropical and subtropical areas, with notable relevance in Brazil. Predominantly cultivated in the North and Northeast regions of the country, especially by small-scale farmers, cowpea is a crucial source of low-cost protein and carbohydrates. It plays a strategic role in food security and provides essential nutrients to vulnerable populations, particularly those in the Brazilian Northeast region [1,2].

In 2020, global cowpea production reached approximately 7.4 million tons, cultivated over 12.6 million hectares, resulting in an average yield of 0.587 t ha^−1^ [3]. Brazil is the third-largest cowpea producer worldwide, with the North and Northeast regions accounting for 94.5% of the cultivated area. In the 2023/2024 growing season, the national average yield was 0.449 t ha^−1^. Despite having a smaller cultivated area—around 5.4 thousand hectares—the Central-West region led in productivity, reaching 1.1 t ha^−1^, particularly in the state of Mato Grosso [4]. During the same period, the North region achieved an average yield of 0.869 t ha^−1^, with the states of Amazonas and Tocantins standing out, cultivating a combined area of 9.4 thousand hectares. In the Northeast, the average yield was 0.425 t ha^−1^, with Maranhão representing the leading producer (0.541 t ha^−1^) and Piauí having the largest cultivated area (186 thousand hectares) [4]. Yields are even lower in the semi-arid region of the Northeast, reaching only 0.289 t ha^−1^ [5].

The main factors contributing to the low cowpea productivity in the Northeast—particularly in the semi-arid region—include limited adoption of research-based technologies, abiotic stresses, and the cultivation of low-yielding varieties [6,7]. Nonetheless, cowpea possesses considerable genetic diversity, especially among traditional varieties, which have been cultivated for generations and exhibit greater adaptation to local environmental conditions compared to improved cultivars.

These varieties are preserved by seed custodians in traditional rainfed farming systems, such as those in the Brazilian semi-arid region, and possess genes that confer tolerance to local environmental constraints [7]. They are often preferred by farmers who contend with the challenges of the semi-arid environment [8].

The semi-arid region encompasses a vast portion of northeastern Brazil and is characterized by challenging edaphoclimatic conditions. Rainfall is poorly distributed, with precipitation concentrated in short, high-intensity periods, contributing to regional water scarcity. These conditions are compounded by high temperatures and low relative humidity [9,10,11]. Under such limitations, irrigation becomes an essential practice for agriculture. However, much of the water available for irrigation contains high concentrations of soluble salts—e.g., desalination by-products—which, although they expand water availability, pose risks of soil salinization and osmotic stress for plants [12,13].

The reuse of saline effluent from desalination systems is particularly relevant in semi-arid communities due to the scarcity of freshwater. In addition to minimizing the environmental impacts associated with the improper disposal of these effluents, their use in irrigation may represent a sustainable strategy when combined with appropriate irrigation and drainage management practices, thereby reducing excessive salt accumulation in the soil. Addressing this approach is essential, as the reuse of desalination brine contributes to regional water sustainability, while simultaneously mitigating the negative effects of direct environmental discharge and promoting social benefits, such as increased access to water for agricultural activities. Thus, management strategies that reconcile agricultural productivity with natural resource conservation are indispensable in semi-arid areas.

Cowpea is moderately tolerant to salinity, with irrigation water salinity thresholds of 3.3 dS m^−1^ and 4.9 dS m^−1^ for the soil saturation extract [14]. Under salt stress, cowpea plants exhibit stomatal closure, reduced transpiration, internal CO_2_ concentration, photosynthesis, dry matter accumulation, nodulation, and leaf area [15,16]. Nevertheless, saline water is commonly used in semi-arid agriculture due to the lack of better-quality water sources.

This reality poses a significant challenge for local agriculture, making saline water often the only available option. The most critical changes induced by salinity in irrigation water include alterations in osmotic potential and ionic toxicity, which result in overall reductions in plant growth [15,16,17]. To optimize the use of saline water and minimize its adverse effects, it is necessary to adopt sustainable management practices and technologies that can mitigate its harmful impacts.

Irrigation water salinity above the tolerance threshold of cowpea reduces plant height and leaf number, significantly affecting plant physiology, yield, and biomass production [18]. Salt stress leads to osmotic imbalance and water-related disorders in the plant, limiting gas exchange and resulting in decreased efficiency of the photosynthetic apparatus. Therefore, it is essential to implement strategies that enhance these physiological parameters, such as the use of stress-mitigating substances and fertilization practices that promote healthy plant growth even under high electrical conductivity conditions.

Although several strategies have been proposed to mitigate the effects of salt stress in plants—including the application of compounds such as salicylic acid [19,20,21] and silicon [22]—other approaches have focused on fertilization with macronutrients such as nitrogen, potassium [23], and phosphorus [17]. However, the specific role of magnesium (Mg) in this context remains unexplored. Magnesium plays critical roles in plant biochemical processes, being essential for photosynthesis, enzyme activation, and the stability of cellular membranes [24,25,26,27].

There is a growing interest in understanding how foliar magnesium fertilization can help cowpea plants combat the challenges posed by salinity, yet research on this topic remains limited. Unveiling the potential benefits of magnesium could be key to enhancing cowpea resilience under salt stress. Thus, we hypothesize that foliar Mg application may be a viable strategy to mitigate the detrimental effects of high salt concentrations in irrigation water on cowpea cultivation. Therefore, we evaluated the potential of foliar Mg fertilization to alleviate salt stress effects on growth, yield components, and leaf gas exchange in two traditional cowpea varieties, ‘Pingo de Ouro’ and ‘Costela de Vaca’.

## 2. Results

### 2.1. Growth and Yield Components

There was a significant interaction between salinity and cowpea variety for the variables of main stem length (MSL) (*p* ≤ 0.05), shoot dry mass (SDM) (*p* ≤ 0.05), and grain yield per plant (GY) (*p* ≤ 0.001) (Table 1). An interaction was also observed between variety and Mg dose for MSL (*p* ≤ 0.05), and between salinity and Mg dose for GY (*p* ≤ 0.05). Moreover, a three-way interaction (salinity × variety × dose) was detected for stem diameter (SD) (*p* ≤ 0.05). A significant isolated effect of salinity was observed on the number of leaves (NL) (*p* ≤ 0.001), and the cowpea variety had an isolated effect on the electrical conductivity of the soil saturation extract (ECse) (*p* ≤ 0.05). In addition, variety (*p* ≤ 0.001) and Mg dose (*p* ≤ 0.10) showed isolated effects on NL.

**Figure 1:**
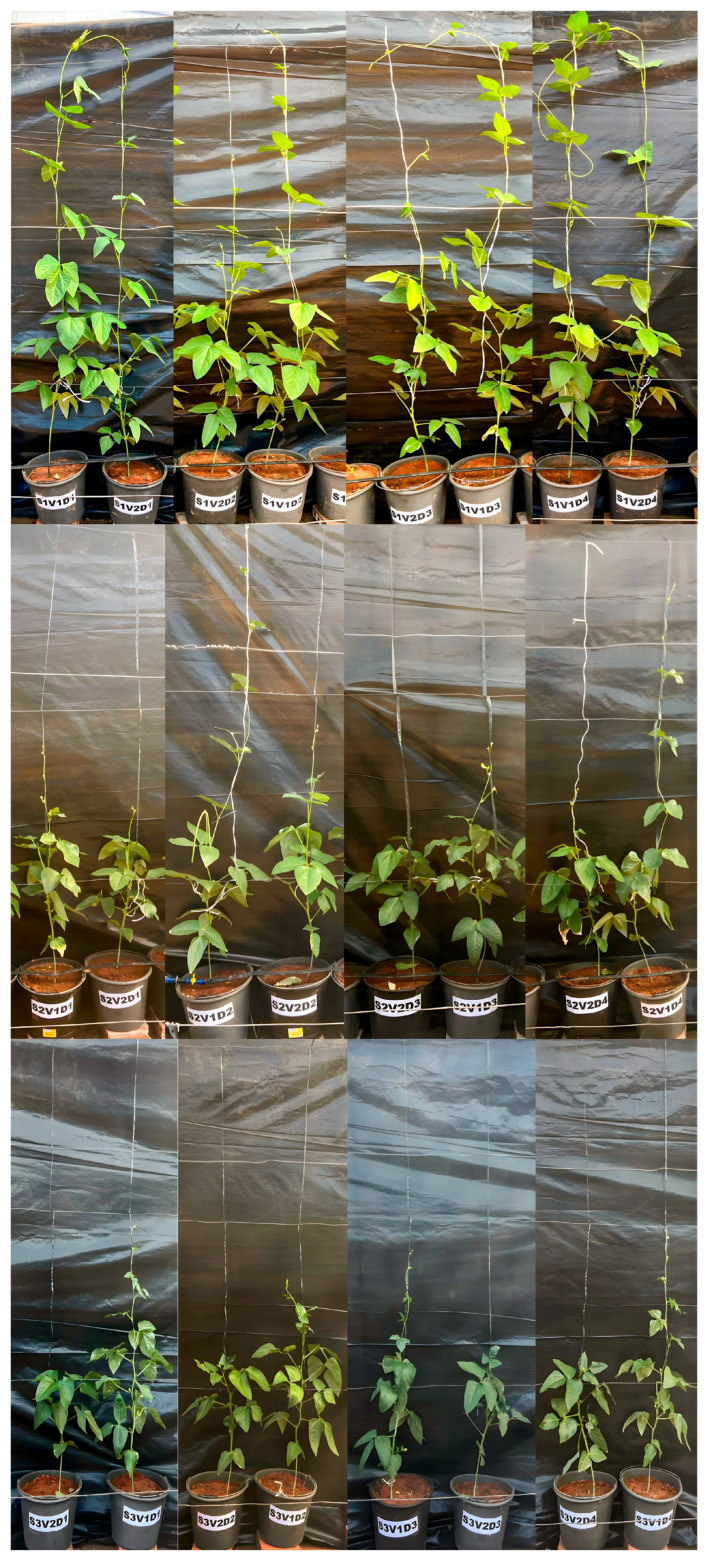
Cowpea plants (V1—‘Pingo de Ouro’, V2—‘Costela de Vaca’) under salinity levels of irrigation water (S1—0.54 dS m^−1^, S2—3.50 dS m^−1^, S3—5.00 dS m^−1^) and foliar Mg doses (D1 = 0 mL L^−1^, D2 = 1 mL L^−1^, D3= 2 mL L^−1^, D4 = 3 mL L^−1^).

Main stem length (MSL) did not differ significantly between V1 and V2 under salinity level S1, with V1 showing a mean of 234.58 cm and V2 of 216.60 cm (Table 2, Figure 1). Under the other salinity levels, significant differences were observed between varieties, with V1 exhibiting higher values than V2 under all conditions. In S2, both varieties experienced a reduction in MSL compared to the control (S1), with V1 decreasing by 21.09% and V2 by 39.41%. Under S3, V1 showed a 44.00% reduction, while V2 exhibited a more pronounced reduction of 68.03% relative to S1. Additionally, a general reduction in MSL was observed with increasing salinity levels.

Shoot dry mass (SDM) did not differ significantly between varieties under salinity level S1, with V1 showing a mean of 31.98 g and V2 of 34.11 g, nor under S2, where V1 had a mean of 15.76 g and V2 of 15.81 g. A significant difference was observed under salinity level S3, with V1 reaching 11.84 g and V2 9.34 g (Table 2). Compared to the control (S1), both varieties showed a reduction in SDM under S2: 50.72% for V1 and 53.65% for V2. Under S3, reductions were even more pronounced, with V1 decreasing by 62.98% and V2 by 72.61%. Overall, both varieties exhibited significantly lower SDM values as salinity increased. No significant differences were found in main stem length (MSL) between Mg doses for either variety (Table 2). However, when comparing the varieties, V1 exhibited higher MSL values than V2 at Mg doses of 0, 1, and 3 mL L^−1^, with increases of 39.82%, 21.23%, and 14.36%, respectively.

Stem diameter (SD) in V1 showed no significant differences among Mg doses under salinity levels S1 and S3. Under S2, however, the 1 mL L^−1^ dose resulted in the highest mean (8.70 mm), differing significantly from the other doses. In V2, no significant differences were observed among Mg doses under S1 and S2 (Table 3). Within each salinity level, V1 showed no differences among doses, except under S2, where the 1 mL L^−1^ dose resulted in a significantly higher SD than under S3. In V2, differences were observed only at the 1 and 3 mL L^−1^ doses. At these doses, mean values under S1 and S2 did not differ but were significantly higher than under S3. Comparing varieties, V2 outperformed V1 at 0 mL L^−1^ under S2 and S3, at 2 mL L^−1^ under S2, and at 3 mL L^−1^ under both S1 and S2.

The number of leaves (NL) decreased as salinity levels increased. The mean NL under S1 (11.95 leaves) was significantly higher than those observed under S2 and S3. S2 exhibited a 22.76% reduction compared to S1, while S3 showed a 36.82% reduction (Table 4). A significant difference in NL was also observed between the two varieties, with V1 exhibiting a 10.02% higher mean than V2 (Table 4). Number of leaves varied significantly with foliar Mg doses. The highest NL mean (10.13 leaves) was recorded at 0 mL L^−1^, while the lowest (9.13 leaves) was observed at 2 mL L^−1^ (Table 4).

A significant interaction between variety and dose was observed for grain yield per plant (GY) (Table 5). For variety V1, GY was significantly higher under S1 (27.12 g plant^−1^) but declined sharply as salinity increased, reaching 15.74 g plant^−1^ under S2 and 9.275 g plant^−1^ under S3. This represented a 65.8% reduction compared to S1, indicating high sensitivity of V1 to increasing salinity. Although V2 also exhibited a downward trend in yield with increasing salinity, it was less affected than V1. Under S1, V2 had the highest yield (31.785 g plant^−1^), 17.2% greater than V1. Under S2, yield dropped to 17.05 g plant^−1^, closely matching V1, with no significant difference. Under S3, V2 yielded 9.63 g plant^−1^, representing a 69.7% reduction from S1, but not significantly different from V1 under high salinity.

Thus, V2 had a higher yield than V1 under low salinity (S1), but this difference diminished as salinity increased, with no significant differences under S2 and S3. These results indicate that while V2 performs better under favorable conditions, both varieties respond similarly under moderate and high salinity. In summary, V1 is more sensitive to salinity than V2; however, both exhibit significant yield reductions under increased salinity, with the most severe effect observed at S3 (Table 5).

A significant interaction was observed between salinity and Mg doses for the variable GY (Table 5). Under S1, yield was the highest, regardless of the applied Mg dose. The dose of 0 mL L^−1^ resulted in a mean yield of 30.57 g plant^−1^, with no significant difference from the 1 mL L^−1^ (30.04 g plant^−1^) and 2 mL L^−1^ (29.41 g plant^−1^) doses. However, the 3 mL L^−1^ dose caused a slight reduction in yield, averaging 27.79 g plant^−1^, which was significantly lower than that of the 0 mL L^−1^ dose. This reduction may indicate a negative effect of higher Mg doses under low salinity conditions.

At salinity level S2, yield was significantly reduced at all Mg doses. The 1 mL L^−1^ dose yielded the highest production at 18.29 g plant^−1^, outperforming the other doses. In contrast, the 2 mL L^−1^ dose resulted in the lowest yield (14.34 g plant^−1^), which was significantly lower than the 0 and 1 mL L^−1^ doses. This suggests that under moderate salinity, foliar application of 1 mL L^−1^ Mg may exert a beneficial effect, while higher doses may be detrimental (Table 5).

At S3, yield was drastically reduced, with means ranging from 8.85 to 9.95 g plant^−1^, with no significant differences among doses. This indicates that, under high salinity, Mg application—regardless of dose—had no significant effect on yield. High salinity appears to be the main limiting factor, suppressing the potentially beneficial effects of Mg. Overall, yield decreased with increasing salinity, regardless of Mg dose. Under S1, yield was approximately 73% higher than under S2 and nearly three times higher than under S3, confirming cowpea’s sensitivity to increased salinity. Under S2, a more favorable response was observed at the 1 mL L^−1^ Mg dose, whereas under S3, Mg application provided no significant benefits (Table 5).

### 2.2. Leaf Gas Exchange, Photosynthetic Pigments, and Foliar Magnesium Content

An interaction between salinity and variety was observed for chlorophyll *a* (*p* ≤ 0.10), chlorophyll *b* (*p* ≤ 0.01), and total chlorophyll (*p* ≤ 0.05) (Table 6). An interaction between salinity and Mg dose was detected for stomatal conductance (*gs*) (*p* ≤ 0.10), internal CO_2_ concentration (*Ci*) (*p* ≤ 0.10), and instantaneous water use efficiency (WUE*i*) (*p* ≤ 0.05). A three-way interaction among salinity, variety, and Mg dose was found for CO_2_ assimilation rate (AN) (*p* ≤ 0.05) and instantaneous carboxylation efficiency (CE*i*) (*p* ≤ 0.05). A significant effect of salinity alone was found for transpiration rate (E) (*p* ≤ 0.001) and leaf temperature (Tl) (*p* ≤ 0.001). An isolated effect of variety was found for internal CO_2_ concentration (*Ci*) (*p* ≤ 0.05) (Table 6).

Regarding the net CO_2_ assimilation rate (A*_N_*), for variety V1, the highest means were observed under S1, except for the 2 mL L^−1^ dose, where S1 did not differ significantly from S2. For variety V2, the highest means were also recorded under S1. When comparing the performance of the varieties, no significant differences were observed regardless of the dose or salinity level (Table 7). For variety V1, the CO_2_ assimilation rate (A*_N_*) did not differ significantly among Mg doses under S1. Under S2, the highest A*_N_* mean was obtained with the 2 mL L^−1^ dose (15.856 µmol m^−2^ s^−1^), which was significantly higher than the other doses. Under S3, the highest A*_N_* mean was observed with the 0 mL L^−1^ Mg dose (12.374 µmol m^−2^ s^−1^). For variety V2, Mg doses did not differ significantly under S1 and S2. However, under S3, the 1 mL L^−1^ dose showed a significantly higher A*_N_* mean (11.765 µmol m^−2^ s^−1^) than the others.

When analyzing the instantaneous carboxylation efficiency (ICE) across salinity levels within variety V1, the highest mean values were observed under salinity S1, except at the 2 mL L^−1^ Mg dose, which did not differ significantly from salinity S2 (Table 7). For variety V2, the highest ICE values were also recorded under salinity S1. Comparing varieties, V1 exhibited higher ICE than V2 under salinity S2 at Mg doses of 0 and 2 mL L^−1^. V1 also surpassed V2 under salinity S1 at the 3 mL L^−1^ dose. Within V1, ICE did not vary significantly with foliar Mg doses under salinity S1. Under S2, the highest mean ICE (0.0816) was recorded at the 2 mL L^−1^ dose, which was significantly higher than that observed at 1 mL L^−1^. Under salinity S3, the highest ICE was observed in the absence of Mg (0 mL L^−1^), surpassing the 3 mL L^−1^ dose by 109.23%. For V2, foliar Mg application had no significant effect on ICE under any salinity level (S1, S2, or S3).

Transpiration (*E*) was highest under salinity S1, reaching 2.9472 mmol m^−2^ s^−1^. Compared to the control (S1), transpiration decreased by 14.68% under S2 and by 20.93% under S3 (Table 8). Leaf temperature (Tl) increased with rising salinity levels. The mean Tl under S1 was significantly lower than those under S2 and S3, with increases of 2.42% and 3.40%, respectively. The highest Tl was recorded under S3, reaching 26.46 °C (Table 8).

When comparing salinity levels for stomatal conductance (gs), the highest gs means were observed under salinity S1 across all foliar Mg doses, except at 2 mL L^−1^, which did not differ from S2 (Table 9). Within salinity level S1, gs did not vary significantly with Mg doses. Similarly, no differences were observed among Mg doses under salinity level S2. Under salinity S3, the highest gs value occurred at 0 mL L^−1^, with a 21.45% reduction observed at the 3 mL L^−1^ dose.

The internal CO_2_ concentration (*Ci*) did not differ significantly with foliar Mg doses under salinities S1 and S2. However, under salinity S3, the 3 mL L^−1^ dose resulted in the highest mean value (273.529 µmol mol^−1^), significantly surpassing the other doses. Across salinity levels, the highest *Ci* means were consistently observed under S3 for all Mg doses (Table 9). Instantaneous water use efficiency (*WUEi*) showed no significant differences among Mg doses under salinities S1 and S2. Under salinity S3, the 0 and 1 mL L^−1^ doses exhibited similar WUE*i* values, both significantly higher than that of the 3 mL L^−1^ dose. Regarding salinity effects, at Mg doses of 0, 1, and 3 mL L^−1^, the highest IWUE values occurred under S1. At the 2 mL L^−1^ dose, IWUE values under S1 and S2 were statistically similar (Table 9).

The internal CO_2_ concentration (*Ci*) differed between genotypes, with variety V2 exhibiting a 3.84% higher mean than variety V1 (Table 10).

Chlorophyll *a* (Chl *a*) content showed no significant differences across salinity levels for variety V1. In contrast, for variety V2, the highest Chl *a* value was observed under salinity S3 (32.71 ICF) (Table 11). No significant differences in Chl *a* were found between varieties under salinity S1, with V1 and V2 presenting mean values of 31.31 and 31.11 ICF, respectively. Under salinity S2, both varieties showed similar values (V1: 31.94 ICF; V2: 31.99 ICF). Under salinity level S3, V1 had a mean value of 31.50 ICF, while V2 presented a significantly higher mean of 32.71 ICF.

Chlorophyll *b* (Chl *b*) content did not differ across salinity levels for variety V1. However, for variety V2, a significant increase was observed under salinity level S3, with a mean value of 25.44 ICF. No significant differences in Chl *b* were found between varieties under salinity levels S1 and S2. Under salinity level S3, V1 showed a mean of 22.39 ICF, while V2 presented a significantly higher mean of 25.44 ICF (Table 11).

Regarding salinity levels, total chlorophyll (Chl *t*) content was highest under salinity levels S2 and S3 (Table 11). For variety V2, the highest Chl *t* value was recorded under salinity S3, with a mean of 58.94 ICF. No significant differences in Chl *t* were observed between varieties under salinity level S1 (V1: 51.76 ICF; V2: 50.52 ICF) or S2 (V1: 55.13 ICF; V2: 55.03 ICF). Under salinity level S3, V2 exhibited a higher mean (58.94 ICF) compared to V1 (54.35 ICF).

Leaf magnesium (Mg) content in cowpea was significantly affected by irrigation water salinity. Under the lowest electrical conductivity level (S1—0.54 dS m^−1^), plants exhibited the highest foliar Mg content, with a mean of 6.231 g kg^−1^ (Table 12). There was a significant reduction in the Mg contents with the increase in salinity (S2—3.50 dS m^−1^ and S3—5.00 dS m^−1^), with respective values of 5.026 g kg^−1^ and 4.486 g kg^−1^ (Table 12).

**Figure 2:**
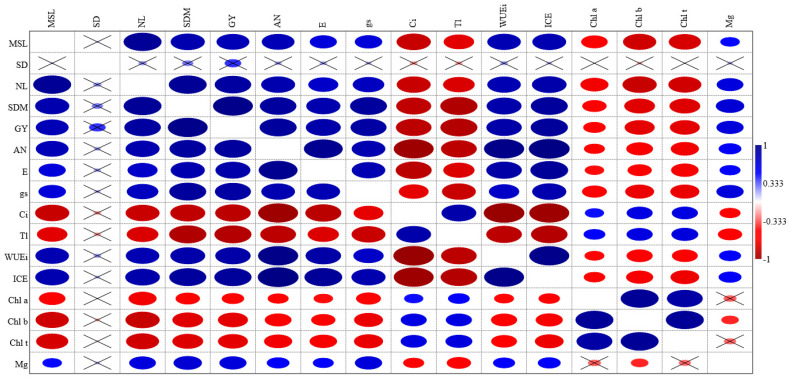
Person (r) correlation analysis for cowpea varieties under salinity levels and foliar magnesium doses.

Person’s correlation analysis (r) shows a strong and significant correlation between grain yield per plant (GY) and main stem length (MSL), number of leaves (NL), shoot dry mass (SDM), net CO_2_ assimilation rate (*A_N_*), transpiration (*E*), stomatal conductance (*gs*), instantaneous water use efficiency (*WUEi*), instantaneous carboxylation efficiency (*CEi*), and leaf magnesium content (Mg). We also found a strong and significant correlation between leaf magnesium content (Mg) and MSL, NL, SDM, *A_N_*, *E*, *gs*, *WUEi*, and *CEi* (Figure 2).

## 3. Discussion

Identifying effective alternatives for irrigating crops with water that have high salt concentrations is vital for addressing food insecurity, particularly in semi-arid regions where fresh, high-quality water is increasingly scarce. Innovative research focused on enhancing crop resilience and performance in saline water irrigation—particularly through the application of substances that alleviate salt stress —plays a crucial role in developing sustainable agricultural practices. These strategies maximize crop yields in challenging conditions and contribute to the overall stability of food supplies in vulnerable communities, especially regarding cowpea production. This research evaluated the potential of foliar magnesium (Mg) fertilization to alleviate the effects of salt stress on growth, yield components, and leaf gas exchange in two traditional cowpea varieties: V1—‘Pingo de Ouro’ and V2—‘Costela de Vaca’. We found that at a salinity level of 3.50 dS m^−1^, a foliar application of 1 mL L^−1^ resulted in the highest yield per plant. For the ‘Costela de Vaca’ variety, CO_2_ assimilation was highest with 2 mL L^−1^ of Mg at the same salinity level (3.50 dS m^−1^), while the ‘Pingo de Ouro’ variety showed the greatest CO_2_ assimilation with 1 mL L^−1^ of Mg at a higher salinity of 5.00 dS m^−1^. The ‘Pingo de Ouro’ variety demonstrated greater tolerance to salt stress compared to ‘Costela de Vaca’. Overall, foliar Mg fertilization appears to be a promising strategy for mitigating the effects of salt stress in cowpea, particularly for the ‘Pingo de Ouro’ variety. While magnesium plays a vital role in alleviating salt stress in plants; however, its effectiveness can differ significantly depending on the specific plant variety and the salinity in the irrigation water. This variability highlights the need to customize magnesium dosage for each crop, emphasizing their optimal growing conditions.

Our findings show that the doses of Mg influenced plant yields differently depending on the salinity levels. Under salinity S1 (0.54 dS m^−1^), Mg application had minimal impact on yield, suggesting that under low-salinity conditions, Mg supplementation does not confer significant benefits to yield. The lack of response in this scenario may be attributed to the plant’s ability to maintain nutritional and osmotic balance without additional Mg. In low-salinity environments, osmotic stress is less pronounced, allowing for adequate nutrient uptake. Additionally, photosynthesis remains relatively unaffected, as stomatal conductance and water-use efficiency are preserved, supporting energy production and carbon assimilation [28]. Therefore, Mg supplementation becomes less critical under such conditions, as photosynthetic and metabolic functions remain stable. However, the 3 mL L^−1^ dose exhibited a slight reduction in yield, which may indicate that, in the absence of salt stress, higher Mg doses could be detrimental, potentially due to nutritional imbalance or mild toxicity. Under salinity level S2 (3.50 dS m^−1^), the 1 mL L^−1^ dose resulted in the highest yield, indicating that this dose was most effective in mitigating moderate salt stress. This effect may be linked to Mg’s role in photosynthesis and enzyme activation, helping maintain metabolic processes under adverse conditions [29]. In contrast, the 2 mL L^−1^ dose yielded the lowest production, suggesting that higher Mg concentrations under moderate salinity may exacerbate osmotic stress, thus reducing yield. Under S3 (5.00 dS m^−1^), the yield was drastically reduced at all Mg doses, with no significant differences between them. This indicates that under high salinity, osmotic stress overrides the beneficial effects of foliar Mg application. Elevated salt concentrations in the soil likely impaired Mg uptake, limiting its contribution to yield improvement. Additionally, sodium-induced ionic toxicity may have disrupted key physiological processes such as nutrient transport and photosynthesis, rendering Mg application ineffective.

Regarding the two varieties studied, Praxedes et al. [30] reported higher yields, particularly in variety V2, with values substantially above those observed in the present study. A likely explanation is the difference in fertilization regimes: the referenced study employed the full recommended dose of fertilizers [31], whereas this study used only 50% of that dosage. Reduced fertilization may have limited the availability of essential nutrients such as nitrogen, phosphorus, and potassium—critical for vegetative and reproductive growth [32]. This limitation likely constrained photosynthetic capacity, reducing biomass production and, consequently, yield. In contrast, full fertilization in the previous study ensured greater nutrient availability, promoting plant growth and significantly enhancing yield.

Salinity exposure significantly affected plant development, as evidenced by reduced main stem length (MSL). In variety V2, the reduction was more pronounced, indicating greater sensitivity to high salinity levels and a consequent impairment in growth. Conversely, variety V1 also exhibited reduced MSL under the same conditions, but to a lesser extent, suggesting slightly greater salt stress tolerance compared to V2. These results align with those of Andrade et al. [33], who found that MSL decreased by an average of 46.25% under salinity of 5.1 dS m^−1^ compared to a control level of 0.6 dS m^−1^ when evaluating salt-tolerant cowpea varieties. These findings reinforce that V1 is more tolerant to salt stress in terms of stem length than V2. The reduction in MSL is primarily attributed to the decreased soil osmotic potential, which increases water retention forces, impeding water uptake and cell turgor, thereby negatively affecting cell elongation and division rates [34]. With regard to Mg doses, although no significant effect on MSL was observed, variety V1 consistently showed higher means across nearly all treatments. This suggests a potential advantage of Mg foliar application in managing salinity stress for this variety.

Salt stress led to an average reduction of 10% in stem diameter (SD), consistent with observations by Oliveira et al. [35], who reported a similar decrease in variety V1 under 4.0 dS m^−1^ salinity compared to the control (0.35 dS m^−1^). This reduction is likely a plant response to the limited water uptake caused by osmotic stress, which diminishes sap flow through conductive tissues [36]. Moreover, sodium-induced ionic toxicity can inhibit the uptake of several nutrients, including magnesium, which plays a key role in the transport of photoassimilates via the plant’s vascular system. Reduced Mg absorption may have contributed to the decline in SD. This is supported by the physiological effects of Mg deficiency, which disrupts carbon metabolism and, secondarily, reduces stem thickness [37]. Under salinity level S2, foliar application of 1 mL L^−1^ Mg led to an 11.82% increase in SD for variety V1 compared to the untreated control, suggesting efficient Mg uptake and utilization by this genotype. In contrast, none of the Mg doses significantly affected SD in variety V2, implying that lower doses may be more appropriate for this variety or that additional strategies beyond Mg supplementation are required to mitigate salt stress under moderate salinity. At the highest salinity level (S3), SD tended to decline across all Mg treatments, particularly for variety V2. These findings underscore the importance of considering the interaction between salinity level, genotype, and Mg dose when evaluating stem growth in cowpea.

Salinity at S2 caused a 22.76% reduction in the number of leaves (NL) compared to S1, while salinity at S3 led to a more pronounced decline of 36.82%. Similarly, Oliveira et al. [35] observed a 17.1% reduction in NL in cowpea variety V1 under 4.0 dS m^−1^ salinity relative to the control (0.35 dS m^−1^). In another study, Praxedes et al. [30] reported an average 50% reduction in the NL of cowpea under 4.5 dS m^−1^ compared to 0.5 dS m^−1^. Such variation in NL response among studies is likely due to the high genetic diversity of traditional cowpea varieties, which results in different salinity tolerance levels. Regarding foliar Mg doses, no significant differences were observed between varieties. However, when analyzed individually, variety V1 exhibited higher NL values than V2, primarily due to its greater main stem length (MSL), which correlates with higher leaf production.

Overall, Mg doses had no significant effect on instantaneous water use efficiency (WUE*i*). However, under high salinity, the 3 mL L^−1^ dose resulted in the lowest WUE*i* mean. This finding suggests that high Mg concentrations under salt stress may have adverse effects, intensifying osmotic stress and ionic toxicity, which compromise stomatal regulation and photosynthesis. Thus, selecting an appropriate Mg dose is critical to optimizing WUE*i* under saline conditions and avoiding excess application that could impair plant performance. In both varieties, salinity level S2 caused an average reduction of 31.6% in net photosynthesis compared to the control (S1). This decline may be attributed to multiple salt stress-related factors. High salt concentrations induce osmotic stress, leading to stomatal closure and reduced CO_2_ uptake. Additionally, salinity can damage chloroplasts, affecting photosystem II efficiency and compromising the photosynthetic capacity of the plant [38].

Regarding CO_2_ assimilation (A*_N_*) in variety V1 under a salinity level of S2, a foliar application of 2 mL L^−1^ magnesium (Mg) resulted in an 11.8% increase in A*_N_* compared to the 0 mL L^−1^ treatment. These findings indicate that applying 2 mL L^−1^ of Mg through foliar fertilization can help alleviate salt stress in variety V1. Other doses of magnesium did not show significant differences in effects. Magnesium deficiency, often induced by sodium toxicity, can reduce the activity of the RuBisCO enzyme, impairing CO_2_ assimilation [39,40]. Mg also plays a key role in regulating stomatal opening and closing [41] directly influencing gas exchange and transpiration. Foliar Mg application circumvents ionic competition in the rhizosphere by allowing direct absorption through leaves, improving ionic balance and osmotic adjustment, and consequently enhancing photosynthetic efficiency [42,43]. For variety V2, the 1 mL L^−1^ Mg dose under salinity level S3 resulted in the highest Aₙ. While the 2 mL L^−1^ dose may have supplied enough Mg to compensate for ionic and osmotic stress in V1, variety V2 may exhibit higher Mg-use efficiency for photosynthesis, making the lower dose (1 mL L^−1^) sufficient to optimize A*_N_* even under severe salinity (S3). These findings indicate that excess Mg may not be beneficial and could cause nutritional imbalances. In V2, 1 mL L^−1^ appears to be the optimal concentration, offering the benefits of Mg without adverse effects. Thus, photosynthetic efficiency varies with genotype and salt stress intensity, leading to different responses to foliar Mg application.

With regard to internal CO_2_ concentration (Ci), the V1 variety showed that applying 3 mL L^−1^ of magnesium at salinity level S3 resulted in a higher internal CO_2_ concentration compared to other doses at this salinity level. This outcome can be attributed to various factors related to the role of magnesium in photosynthesis and the plant’s responses to salt stress. Salt stress can reduce photosynthetic efficiency, leading to CO_2_ accumulation in the leaves due to the decline in carbon assimilation rates. As magnesium is essential for the activation of RuBisCO—the enzyme responsible for CO_2_ fixation in the Calvin cycle—ensuring an adequate Mg supply can help maintain the enzyme’s efficient functioning, even under salt stress conditions [29]. Although salinity level S3 represents a condition of severe stress for plants, the application of 3 mL L^−1^ of Mg may have significantly alleviated the osmotic and ionic stress, resulting in a higher *Ci*. This suggests better maintenance of stomatal opening and increased availability of CO_2_ for photosynthesis. The application of 3 mL L^−1^ of Mg in variety V1 under S3 salinity conditions led to a higher *Ci* compared to other doses, indicating that this specific Mg concentration may be effective in mitigating the negative effects of salt stress and in improving the plant’s ability to sustain gas exchange and internal CO_2_ availability. However, an increase in *Ci* may also indicate that photosynthesis is not occurring efficiently, possibly due to reduced enzymatic activity [44]. This rise in *Ci* may reflect that, despite the higher availability of CO_2_, photosynthesis is still limited due to the severity of the salt stress, which may impair other components of the photosynthetic process—even with Mg supplementation. Therefore, adjusting Mg doses appropriately according to salinity levels is essential to optimize growth and photosynthetic efficiency in cowpea.

The Instantaneous Carboxylation Efficiency (ICE) for the variety V2, when treated with a 1 mL L^−1^ Mg dose under salinity S2, was higher than for the other doses. This may be attributed to V2’s superior ability to utilize magnesium, making the lower dose sufficient to optimize ICE. Higher magnesium doses could lead to nutritional imbalances or even toxicity in V2, while the 1 mL L^−1^ dose seems to strike an ideal balance, providing benefits without any adverse effects. For variety V1, the 2 mL L^−1^ Mg dose was the most effective under salinity S2, likely due to this variety’s need for a greater amount of Mg to counteract the negative effects of salinity and maximize carboxylation. Magnesium is essential for activating the RuBisCO enzyme, which plays a key role in CO_2_ fixation in the Calvin cycle, thereby increasing photosynthetic efficiency and ICE. Although an increase in internal CO_2_ concentration may initially seem unfavorable, when associated with improved ICE, it indicates a beneficial adjustment. By activating RuBisCO and enhancing CO_2_ assimilation, Mg helps stabilize cell membranes and optimize photosynthetic processes under salt stress [45,46]. Thus, even with higher CO_2_ concentration, the plant efficiently uses the gas, favoring carbohydrate production and growth [47], mitigating the adverse effects of salt stress and maintaining photosynthetic efficiency [48]. These findings show that the response of instantaneous carboxylation efficiency to Mg varies between cowpea varieties under saline conditions. While variety V1 benefits more from the 2 mL L^−1^ Mg dose under S2 salinity, variety V2 achieved higher ICE with the 1 mL L^−1^ dose. These findings highlight the importance of adjusting Mg supplementation according to plant variety and stress conditions to optimize photosynthesis and carboxylation.

In summary, our findings indicate that foliar application of magnesium is a promising strategy for enhancing salt stress tolerance in cowpea. This is particularly relevant for farmers in semi-arid regions where salinity is a persistent challenge. This practice can help mitigate the harmful effects of salinity, allowing plants to maintain essential physiological functions such as photosynthesis and pod formation, even in adverse conditions. However, the effectiveness of magnesium application depends on several factors, including the dosage used, the specific variety of cowpea cultivated, and the level of salinity present. For farmers, this means that management practices should be tailored to local conditions and the unique characteristics of the planted varieties. We emphasize that while magnesium application does not completely eliminate the impacts of salinity, it can enhance plant performance, making it a viable option for improving production in sustainable agricultural systems within semi-arid regions.

## 4. Materials and Methods

### 4.1. Experimental Site and Cultivation Conditions

The study was conducted in a greenhouse belonging to the Department of Agronomic and Forestry Sciences (DCAF) of the Federal Rural University of the Semi-Arid Region (UFERSA), in Mossoró, RN, Brazil, from June to August 2023. The municipality is located in the semi-arid region of Northeast Brazil, at the geographical coordinates 5°11′31″ S and 37°20′40″ W, with an elevation of 18 m above sea level. According to the Köppen climate classification, the region has a semi-arid climate (BSh), characterized by highly irregular rainfall [49].

**Figure 3:**
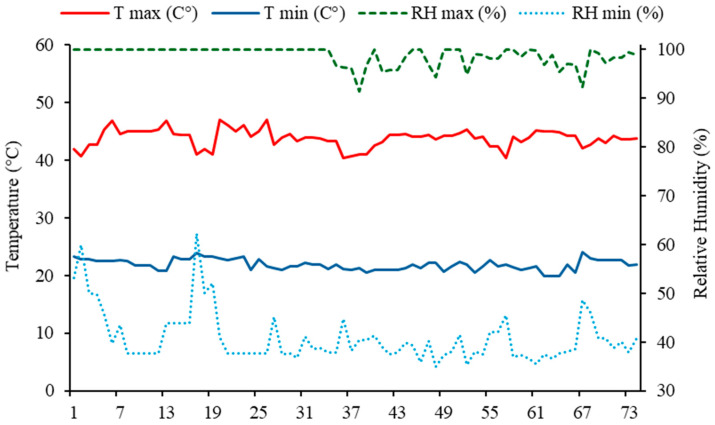
Temperature and relative humidity data recorded during the experiment. T max = maximum temperature. T min = minimum temperature. RH max = maximum relative humidity. RH min = minimum relative humidity.

During the experimental period, climatic conditions inside the greenhouse were monitored. Maximum and minimum air temperatures and relative humidity were recorded using a Digital Thermo-Hygrometer MTH1300 Minipa^®^. Data collection spanned from 14 June to 25 August 2023. Maximum temperatures ranged from 40.3 to 47.0 °C, while minimum temperatures varied between 19.9 and 24.0 °C (Figure 3). The greenhouse is located on UFERSA’s central campus at coordinates 5°12′48″ S and 37°18′44″ W, at an elevation of 18 m above sea level. It is built with galvanized steel arches, measuring 3.5 m in height, 7 m in width, and 18 m in length. The structure is covered with a 150-micron-thick low-density polyethylene film, supplemented with a 50% shade net.

### 4.2. Experimental Design

The experiment followed a randomized complete block design in a 2 × 3 × 4 factorial arrangement with five replicates, totaling 120 experimental units. The factors consisted of two traditional cowpea varieties (V1—‘Pingo de Ouro’; V2—‘Costela de Vaca’), three levels of electrical conductivity of irrigation water (S1—0.54, S2—3.50, and S3—5.00 dS m^−1^), and four foliar Mg doses (0, 1, 2, and 3 mL L^−1^).

Each plot consisted of a 12 dm^3^ polyethylene pot equipped with an external drainage channel near the base. At the start of the experiment, pots were brought to field capacity using irrigation water of the respective salinity level. Foliar Mg applications were performed on the 15th, 32nd, and 38th days after sowing (DAS), corresponding to the V3, V5, and V9 phenological stages of cowpea [36]. The commercial product Forplant^®^ was used for foliar fertilization, with a density of 1.30 g mL^−1^ at 20 °C and a magnesium content of 8%. The applied doses were 0.0, 1.0, 2.0, and 3.0 mL L^−1^. Each plant received 50 mL of solution, sprayed onto the leaves in three applications of 10 mL, 20 mL, and 20 mL, respectively.

### 4.3. Plant Material and Water Sources

Seeds of the traditional cowpea varieties used in this study were obtained directly from local farmers, as these genotypes are widely cultivated in the region. The seeds, harvested in the 2023 growing season, were sourced from seed guardian collections in rural communities in the western region of the state of Rio Grande do Norte, Brazil.

The ‘Pingo de Ouro’ variety is characterized by a semi-prostrate growth habit, with flowering beginning approximately 41 days after sowing and physiological maturity occurring between 71 and 80 DAS. The seeds have a beige seed coat, a 100-seed weight of 19 g, and an average yield of 1118 kg ha^−1^ [50].

‘Costela de Vaca’ plants are considered low-vigor, with average plant height below 37 cm and canopy width under 75 cm [34]. The growth habit is erect to semi-prostrate and indeterminate, with no tendency to twine around support structures. The apical leaflet is sub-globose, with an average length of 98 mm and a width of 69 mm. The variety has compressed short hairs on the stem, light green coloration, membranous texture, and V-shaped marks on the leaflets. Flowering begins approximately 54 to 59 days after emergence, and the white flowers last 3 to 4 days after opening. Mature pods are straw-colored and form an angle between 30° and 90° with the peduncle, which typically bears one or two pods with an average length of 180 mm.

Irrigation water salinity levels were obtained from the brine (reject water) of a reverse osmosis desalination unit that supplies the Jurema settlement, located along road RN-013 between the municipalities of Mossoró and Tibau, Brazil. Dilutions of this brine with local supply water were prepared to achieve the electrical conductivities required for each treatment (Table 13).

Salinity S1 consisted of local tap water. For S2, a mixture was prepared using 55% tap water and 45% saline reject; S3 consisted of 33% S1 and 67% saline reject. These conductivity levels are similar to those of water sources commonly available for irrigation in the Brazilian semi-arid region [51] and follow the relationship between electrical conductivity (EC) and total salt concentration (mmol_c_ L^−1^ = EC × 10) as described by Rhoades et al. [52]. S2 corresponds to the threshold salinity level tolerated by cowpea (3.3 dS m^−1^) [14], whereas S3 was specifically selected to induce salt stress, as it exceeds the crop’s tolerance limit.

### 4.4. Soil Characterization

The soil used was a Dystrophic Red-Yellow Latosol with argic horizon [53], equivalent to an Oxisol [54], collected from the 0–30 cm layer at the Rafael Fernandes Experimental Farm, UFERSA, located in the rural district of Alagoinha, Mossoró-RN, Brazil (5°03′37″ S, 37°23′50″ W). After collection, the soil was air-dried, crushed, sieved through a 2.0 mm mesh, and sampled for physical and chemical analyses.

Soil analysis, saturation extract (Table 14), and water source characterizations (Table 15) were conducted at the Soil, Water, and Plant Laboratory (LASAP) and the Semi-Arid Soil, Water, and Plant Analysis Laboratory (LASAPSA) using the methodologies of Richards [55] for water, and Teixeira et al. [56] for soil and saturation extract analysis.

### 4.5. Fertilization and Crop Management

Macronutrient fertilization followed the recommendations of Novais et al. [31], adjusted to 50% of the standard dose, corresponding to 50 mg N, 150 mg P_2_O_5_, 75 mg K_2_O, 29 mg CaO, 18 mg MgO, and 30 mg SO_4_^2−^ per dm^3^ of soil. Nutrient sources included monoammonium phosphate (61% P_2_O_5_; 12% N), calcium nitrate (18% CaO; 15% N), magnesium sulfate (9% MgO; 11% SO_4_^2−^; 1% K_2_O), potassium sulfate (17.5% SO_4_^2−^; 51.5% K_2_O), and potassium chloride (60% K_2_O). Fertilization was applied via fertigation in nine installments: the first pre-planting (20% of the total dose), followed by eight weekly applications of 10%. Micronutrient fertilization was performed four times during the experiment, with 18-day intervals between applications, using the commercial foliar fertilizer Liqui-Plex Fruit^®^ (Table 15), at a concentration of 3 mL L^−1^, as recommended by the manufacturer. The product has a density of 1.47 g cm^−3^.

During the experiment, weeds that emerged in the pots were manually removed. Whitefly (*Bemisia tabaci*), leafminer (*Liriomyza sativae*), and other pests were controlled through targeted applications of commercial pesticides, applied as needed. At sowing, nine seeds were placed per pot at a depth of 3 cm. The first thinning was performed six days after sowing (DAS), leaving three plants per pot. The second thinning occurred at 15 DAS, reducing to two plants per pot, and the final thinning at 34 DAS left only one plant per pot, prioritizing the most developed plants, with a greater number of leaves, thicker stems and more intense leaf color.

### 4.6. Irrigation Management

The irrigation system consisted of BAV1118-02UC electric pumps (Invensys^®^, 220 V, 60 Hz, 34 W, London, UK) connected to a 100 L reservoir and 16 mm hoses equipped with self-compensating drippers operating at a flow rate of 1.3 L h^−1^. Crop evapotranspiration and irrigation requirements under different salinity levels were estimated using the drainage lysimetry method [57]. Six lysimeters were installed, distributed across the three irrigation water salinity levels (S1, S2, and S3), with one lysimeter for each variety (V1 and V2) per salinity level. Irrigation lines were installed in the greenhouse, each dedicated to a specific salinity level. Water was slowly applied to each lysimeter until field capacity was reached, thus enabling the calculation of irrigation volume per variety and salinity level. When applied water exceeded the soil’s field capacity, the excess was collected and subtracted from the irrigation volume. For each salinity level, field capacity was estimated for both varieties. Irrigation time was then determined based on the average water requirement, calculated using Equation (1).

(1) $$T=\frac{\left(Va-Vd\right)}{Q}60$$

where

**Figure 4:**
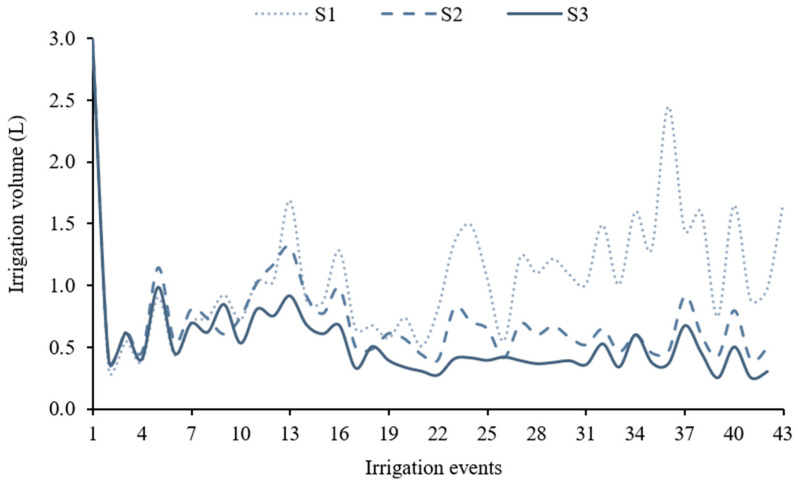
Irrigation volume for each salinity level (S1—0.54, S2—3.50, and S3—5.00 dS m^−1^) throughout the experiment.

*T*—irrigation time (min);

*Va*—Water volume applied to the lysimeters (L);

*Vd*—Water volume drained from the lysimeters (L);

*Q*—Drip rate (L h^−1^);

The total irrigation volume applied throughout the experiment was quantified (Figure 4). Variations in water volume reflect differing crop water requirements in response to soil salinity levels.

Based on the chemical characterization of each irrigation water source and the total volume applied per treatment, the total amount of salts added to the soil was estimated (Table 16) for each salinity level using Equation (2), as proposed by Rhoades et al. [52].

(2) TDS=ECw×640×V

where

*TDS*—Salts dissolved in water (mg L^−1^);

*ECw*—Electrical conductivity of the irrigation water (dS m^−1^);

*V*—Total irrigation volume (L).

This conversion allowed for the estimation of the cumulative salt input into the soil over the course of the experiment, thereby enabling a more accurate assessment of salinity effects on cowpea.

At the end of the experiment, the electrical conductivity of the soil saturation extract (*ECse*) was determined for each pot (Equation (3)), and the mean value was calculated for each salinity level (Table 17). The methodology described by Ayers and Westcot [14] for medium-textured soils was used. A leaching fraction equivalent to 10% of the total irrigation volume was applied to each pot. The leachate was collected, and the electrical conductivity of the drainage water (*ECd*) was measured using a benchtop conductivity meter. The EC values were expressed in dS m^−1^ and adjusted to 25 °C before being applied in Equation (2).

(3) $$ECse=\frac{ECd}{2}$$

### 4.7. Gas Exchange, Photosynthetic Pigments, and Leaf Magnesium Content

Gas exchange measurements were performed at the flowering stage, 38 days after sowing, between 6:00 and 9:00 a.m. Evaluations were conducted on the central leaflets of fully expanded leaves located in the upper third of the plants, using a portable infrared gas analyzer (IRGA), model GFS-3000 Gas-Exchange System (WALZ^®^, Eichenring, Germany), set to a leaf chamber temperature of 25 °C, photosynthetically active radiation (PAR) of 1200 µmol photons m^−2^ s^−1^, and airflow rate of 400 mL min^−1^. The CO_2_ assimilation rate (*A_N_*, µmol m^−2^ s^−1^), transpiration (*E*, mmol H_2_O m^−2^ s^−1^), stomatal conductance (*gs*, mol H_2_O m^−2^ s^−1^), internal CO_2_ concentration (*Ci,* µmol mol^−1^) and leaf temperature (*Tl*, °C) were quantified and used to calculate the instantaneous water use efficiency (*WUEi*) (*A_N_ E*^−1^) [(µmol m^−2^ s^−1^) (mmol H_2_O m^−2^ s^−1^) ^−1^] and the instantaneous carboxylation efficiency (ICE, (*A_N_ Ci*^−1^, [(µmol m^−2^ s^−1^) (µmol mol^−1^)^−1^] [58].

Chlorophyll *a*, chlorophyll *b*, and chlorophyll total Index were also measured by indirect readings using a ClorofiLOG CFL1030 chlorophyll meter (Falker^®^ Automação Agrícola, Maracaju, Brazil), operated according to the instructions of the manufacturer. The device uses photodiodes emitting at 635, 660, and 880 nm to produce the Falker Chlorophyll Index (FCI). Measurements were taken from the same leaflet, with three readings per leaflet in the middle third of the blade to obtain a mean value.

Leaf dry matter was ground in a Willey-type stainless-steel mill and stored in labeled plastic bags. The material was digested in concentrated nitric acid (HNO_3_, 65% p.a.) using a microwave digestion system. Magnesium concentration (Mg^2+^) was determined following EMBRAPA [59], using atomic absorption spectrophotometry (AAS).

### 4.8. Growth and Yield Components

At the reproductive stage (44 DAS), plants were evaluated for main stem length (MSL, cm) using a measuring tape from the plant base to the last leaf insertion; stem diameter (SD, mm), measured 1 cm above the base with a digital caliper; and number of leaves (NL). After pod harvesting, the number of pods per plant and grain yield per plant (GY, g) were recorded. Shoots were collected and dried to constant weight in a forced-air oven at 65 °C to determine shoot dry mass (SDM, g).

### 4.9. Statistical Analysis

Data were subjected to analysis of variance (ANOVA) using the F-test at significance levels of *p* ≤ 0.10, *p* ≤ 0.05, and *p* ≤ 0.01. Means for cowpea varieties were compared using Student’s *t*-test, whereas salinity levels and magnesium doses were compared using Tukey’s test at the same significance levels. Statistical analyses were performed using SISVAR^®^ software, version 5.8 [60]. Pearson correlation analysis was performed using PAST4 software.

## 5. Conclusions

The cowpea variety ‘Pingo de Ouro’ exhibited a significantly higher tolerance to salinity when compared to ‘Costela de Vaca.’ Additionally, foliar fertilization with magnesium emerged as a promising agronomic practice to alleviate the adverse effects of salt stress, particularly for the ‘Pingo de Ouro’ variety. However, the efficacy of magnesium application is contingent upon both the specific variety and the levels of salinity, emphasizing the necessity for dosage adjustments. A magnesium dosage of 2 mL L^−1^ proved more beneficial for ‘Pingo de Ouro,’ whereas ‘Costela de Vaca’ demonstrated improved outcomes with a reduced dosage of 1 mL L^−1^. These results highlight the potential of foliar magnesium application as an effective management strategy in saline environments. Furthermore, they underscore the imperative for additional research aimed at refining and optimizing dosage levels in open field conditions, thereby ensuring maximal effectiveness in enhancing plant health and resilience.

## Figures and Tables

**Table 1:** Summary of the analysis of variance for the variables main stem length (MSL, cm), stem diameter (SD, mm), number of leaves (NL), shoot dry mass (SDM, g), and grain yield per plant (GY, g) of cowpea varieties.

SV	F-Test (Pr > Fc)				
	MSL	SD	NL	SDM	GY
Sal	0.0000 ***	0.0001 ***	0.0000 ***	0.0000 ***	0.0000 ***
Var	0.0000 ***	0.0000 ***	0.0001 ***	0.8717 ^NS^	0.0000 ***
Dose	0.2352 ^NS^	0.5954 ^NS^	0.0523 ^#^	0.2239 ^NS^	0.0063 ***
Sal × Var	0.0317 *	0.1585 ^NS^	0.2355 ^NS^	0.0213 *	0.0001 ***
Sal × Dose	0.1543 ^NS^	0.4857 ^NS^	0.1241 ^NS^	0.1004 ^NS^	0.0234 *
Var × Dose	0.0361 *	0.6241 ^NS^	0.1406 ^NS^	0.1661 ^NS^	0.1171 ^NS^
Sal × Var × Dose	0.3243 ^NS^	0.0481 *	0.6763 ^NS^	0.7621 ^NS^	0.3071 ^NS^
Block	0.0244 *	0.0435 *	0.0015 **	0.0015 **	0.0121 *
CV (%)	24.29	9.4	14.56	18.5	12.40

*** significant at 0.1% (*p* ≤ 0.001); ** significant at 1% (*p* ≤ 0.01); * significant at 5% (*p* ≤ 0.05); # significant at 10% (*p* ≤ 0.10); NS = not significant. Sal—corresponds to the three levels of irrigation water electrical conductivity (0.54, 3.50, and 5.00 dS m^−1^); Var—refers to the two varieties used in the experiment (V1—‘Pingo de Ouro’; V2—‘Costela de Vaca’); Dose—refers to the four foliar Mg doses applied (0, 1, 2, and 3 mL L^−1^); CV—coefficient of variation.

**Table 2:** Main stem length (MSL, cm) and shoot dry mass (SDM, g) of cowpea varieties (V1—‘Pingo de Ouro’, V2—‘Costela de Vaca’) under irrigation water salinity levels (S1—0.54 dS m^−1^, S2—3.5 dS m^−1^, S3—5.00 dS m^−1^) and foliar Mg doses.

Interaction between varieties and irrigation water salinity levels, Standard error, n = 20.				
Salinity	Variety			
	MSL (cm)		SDM (g)	
	V1	V2	V1	V2
S1	234.58 ± 6.86 Aα	216.60 ± 11.36 Aα	31.98 ± 0.87 Aα	34.11 ± 0.96 Aα
S2	185.09 ± 10.01 Bα	131.23 ± 10.00 Bβ	15.76 ± 0.81 Bα	15.81 ± 1.11 Bα
S3	131.35 ± 11.97 Cα	69.25 ± 4.58 Cβ	11.84 ± 0.67 Cα	9.34 ± 0.38 Cβ
Interaction between varieties and foliar Mg doses, Standard error, n = 15.				
**MSL (cm)**				
**Variety**	**Mg doses (mL L^−1^)**			
	**0**	**1**	**2**	**3**
V1	203.78 ± 13.57 αa	174.43 ± 16.46 αa	167.37 ± 16.96 αa	189.10 ± 15.31 αa
V2	122.63 ± 16.42 βa	137.40 ± 19.90 βa	139.80 ± 17.42 αa	156.27 ± 22.03 βa

Means followed by the same uppercase letter in the column do not differ significantly for salinity levels according to Tukey’s test (*p* ≤ 0.05). Means followed by the same Greek letter in the row do not differ significantly between varieties according to Student’s *t*-test (*p* ≤ 0.05). Means followed by the same lowercase letter in the row do not differ significantly for the Mg dose according to Tukey’s test (*p* ≤ 0.05).

**Table 3:** Stem diameter (SD, mm) of cowpea plants according to the interaction between varieties (V1—‘Pingo de Ouro’; V2—‘Costela de Vaca’), salinity levels of irrigation water (S1—0.54 dS m^−1^, S2—3.50 dS m^−1^, S3—5.00 dS m^−1^), and foliar Mg doses (0, 1, 2, 3 mL L^−1^).

Variety	Salinity	Mg Doses (mL L^−1^)			
		0	1	2	3
**V1**	S1	8.28 ± 0.34 Aαa	8.02 ± 0.41 ABαa	8.06 ± 0.35 Aαa	7.42 ± 0.55 Aβa
	S2	7.78 ± 0.24 Aβab	8.70 ± 0.47 Aαa	7.30 ± 0.50 Aβb	7.54 ± 0.30 Aβab
	S3	7.14 ± 0.11 Aβa	7.32 ± 0.45 Bαa	7.32 ± 0.34 Aαa	7.70 ± 0.41 Aαa
V2	S1	8.12 ± 0.25 Aαa	8.70 ± 0.33 ABαa	8.32 ± 0.31 Aαa	8.76 ± 0.16 Aαa
	S2	8.98 ± 0.16 Aαa	8.98 ± 0.43 Aαa	8.74 ± 0.36 Aαa	9.16 ± 0.17 Aαa
	S3	8.40 ± 0.21 Aαa	7.72 ± 0.43 Bαa	8.26 ± 0.45 Aαa	7.52 ± 0.19 Bαa

Means followed by the same uppercase letter in the column do not differ significantly for salinity according to Tukey’s test (*p* ≤ 0.05). Means followed by the same Greek letter in the column do not differ significantly for variety according to Student’s *t*-test (*p* ≤ 0.05). Means followed by the same lowercase letter in the row do not differ significantly for dose according to Tukey’s test (*p* ≤ 0.05). Standard error, n = 5.

**Table 4:** Number of leaves (NL) of cowpea plants under different salinity levels of irrigation water.

Salinity (dS m^−1^)	NL
S1	11.95 ± 0.21 A
S2	9.23 ± 0.28 B
S2	7.55 ± 0.27 C
Variety	**NL**
V1	10.08 ± 0.29 α
V2	9.07 ± 0.32 β
**Mg doses (mL L^−1^)**	**NL**
0	10.13 ± 0.48 a
1	9.47 ± 0.45 ab
2	9.13 ± 0.46 b
3	9.56 ± 0.39 ab

Means followed by the same letter in the column do not differ significantly for salinity according to Tukey’s test (*p* ≤ 0.05). Standard error, n = 40 (Salinity), n = 60 (Variety), and n = 30 (Mg doses).

**Table 5:** Grain yield per plant (GY, g) of cowpea plants according to the interaction between varieties (V1—‘Pingo de Ouro’, V2—‘Costela de Vaca’) and salinity levels of irrigation water (S1—0.54 dS m^−1^, S2—3.50 dS m^−1^, S3—5.00 dS m^−1^).

Salinity	Varieties			
	V1		V2	
S1	27.12 ± 0.75 Aβ		31.785 ± 0.73 Aα	
S2	15.74 ± 0.56 Bα		17.05 ± 0.60 Bα	
S3	9.275 ± 0.37 Cα		9.630 ± 0.33 Cα	
**Salinity**	**Mg doses (mL L^−1^)**			
	**0**	**1**	**2**	**3**
S1	30.57 ± 0.90 Aa	30.04 ± 1.27 Aab	29.41 ± 1.52 Aab	27.79 ± 1.36 Ab
S2	17.46 ± 0.84 Bab	18.29 ± 0.74 Ba	14.34 ± 0.69 Bc	15.49 ± 0.50 Bbc
S3	9.82 ± 0.44 Ca	8.85 ± 0.65 Ca	9.95 ± 0.40 Ca	9.19 ± 0.42 Ca

Means followed by the same uppercase letter in the column do not differ significantly for salinity according to Tukey’s test (*p* ≤ 0.05). Means followed by the same Greek letter in the row do not differ significantly for variety according to Student’s *t*-test (*p* ≤ 0.05). Means followed by the same lowercase letter in the row do not differ significantly for Mg doses according to Tukey’s test (*p* ≤ 0.05). Standard error, n = 20.

**Table 6:** Summary of the analysis of variance for the variables: net CO_2_ assimilation rate (*A_N_*, µmol m^−2^ s^−1^), transpiration (*E*, mmol H_2_O m^−2^ s^−1^), stomatal conductance (*gs*, mol H_2_O m^−2^ s^−1^), internal CO_2_ concentration (*Ci*, µmol mol^−1^), leaf temperature (*Tl*, °C), instantaneous water use efficiency (WUEi = *A_N_/E*, [(µmol m^−2^ s^−1^)/(mmol H_2_O m^−2^ s^−1^)]), instantaneous carboxylation efficiency (CEi = *A_N_*/*Ci*, [(µmol m^−2^ s^−1^)/(µmol mol^−1^)]), chlorophyll *a* (Chl *a*, ICF), chlorophyll *b* (Chl *b*, ICF), total chlorophyll (Chl *t*, ICF), and leaf magnesium content (Mg, g kg^−1^).

FV	F-Test (Pr > Fc)									
	*A_N_*		*E*		*gs*		*Ci*		*Tl*	
**Sal**	0.0000 ***		0.0000 ***		0.0000 ***		0.0000 ***		0.0000 ***	
Var	0.4668 ^NS^		0.3396 ^NS^		0.2429 ^NS^		0.0275 *		0.5465 ^NS^	
Dose	0.0055 **		0.1338 ^NS^		0.0487 *		0.0255 *		0.3142 ^NS^	
Sal × Var	0.4582 ^NS^		0.6328 ^NS^		0.3135 ^NS^		0.7412 ^NS^		0.6601 ^NS^	
Sal × Dose	0.0053 **		0.5718 ^NS^		0.0818 ^#^		0.0521 ^#^		0.3227 ^NS^	
Var × Dose	0.5261 ^NS^		0.9626 ^NS^		0.9554 ^NS^		0.2688 ^NS^		0.8393 ^NS^	
Sal × Var × Dose	0.0456 *		0.5800 ^NS^		0.2137 ^NS^		0.2461 ^NS^		0.9854 ^NS^	
Block	0.0000 ***		0.0000 ***		0.0000 ***		0.0377 *		0.0000 ***	
CV (%)	15.54		14.83		14.17		9.22		1.55	
FV	F-test (Pr (Pr > Fc)									
	*WUEi*	*ICE*		Chl a		Chl b		Chl t		Mg
Sal	0.0000 ***	0.0000 ***		0.0138 *		0.0000 ***		0.0000 ***		0.0000 ***
Var	0.1750 ^NS^	0.0328 *		0.1849 *		0.1818 ^NS^		0.1869 ^NS^		0.9173 ^NS^
Dose	0.1265 ^NS^	0.0267 *		0.7912 ^NS^		0.4203 ^NS^		0.6604 ^NS^		0.5403 ^NS^
Sal × Var	0.6281 ^NS^	0.3104 ^NS^		0.0701 ^#^		0.0064 **		0.0104 *		0.1770 ^NS^
Sal × Dose	0.0191 *	0.0100 *		0.5046 ^NS^		0.4010 ^NS^		0.5103 ^NS^		0.6598 ^NS^
Var × Dose	0.5400 ^NS^	0.1562		0.1293 ^NS^		0.2841 ^NS^		0.3177 ^NS^		0.2987 ^NS^
Sal × Var × Dose	0.3455 ^NS^	0.0134 *		0.9655 ^NS^		0.9249 ^NS^		0.4232 ^NS^		0.6192 ^NS^
Block	0.0012 **	0.0877 ^#^		0.0204 *		0.0898 ^#^		0.0455 *		0.0128 ^NS^
CV (%)	13.71	19.18		4.54		15.03		8.21		15.73

*** Significant at 0.1% (*p* ≤ 0.001); ** significant at 1% (*p* ≤ 0.01); * significant at 5% (*p* ≤ 0.05); # significant at 10% (*p* ≤ 0.10); NS—not significant. Sal—refers to the three electrical conductivity levels of irrigation water (0.54, 3.50, and 5.00 dS m^−1^); Var—refers to the two varieties used in the experiment (V1—‘Pingo de Ouro’, V2—‘Costela de Vaca’); Dose—refers to the four foliar Mg doses applied (0, 1, 2, and 3 mL L^−1^); CV—coefficient of variation.

**Table 7:** Net CO_2_ assimilation rate (A*_N_*, µmol m^−2^ s^−1^) and instantaneous carboxylation efficiency (ICE, [(µmol m^−2^ s^−1^) (µmol mol^−1^)^−1^]) of cowpea varieties according to the interaction between varieties (V1—‘Pingo de Ouro’, V2—‘Costela de Vaca’), irrigation water salinity levels (S1—0.54 dS m^−1^, S2—3.50 dS m^−1^, S3—5.00 dS m^−1^), and foliar Mg doses (0, 1, 2, 3 mL L^−1^).

A*_N_* (µmol m^−2^ s^−1^)					
Variety	Salinity	Mg Doses (mL L^−1^)			
		0	1	2	3
V1	S1	18.43 ± 0.63 Aαa	19.04 ± 1.46 Aαa	17.09 ± 1.86 Aαa	19.86 ± 0.54 Aαa
	S2	14.19 ± 0.83 Bαab	12.05 ± 0.39 Bαb	15.86 ± 0.63 Aαa	12.44 ± 1.23 Bαab
	S3	12.37 ± 1.72 Bαa	10.25 ± 1.35 Bαab	9.54 ± 0.54 Bαab	6.87 ± 0.58 Cαb
V2	S1	19.13 ± 1.26 Aαa	17.33 ± 0.46 Aαa	19.306 ± 0.49 Aαa	18.31 ± 1.02 Aαa
	S2	12.79 ± 1.55 Bαa	13.960 ± 1.08 Bαa	13.06 ± 0.80 Bαa	10.86 ± 1.17 Bαa
	S3	10.52 ± 1.64 Bαab	11.77 ± 1.34 Bαa	9.96 ± 1.42 Bαab	7.57 ± 0.75 Cαb
ICE, [(µmol m^−2^ s^−1^) (µmol mol^−1^)^−1^])					
**Variety**	Salinity	Mg doses (mL L^−1^)			
		0	1	2	3
V1	S1	0.104 ± 0.005 Aαa	0.108 ± 0.007 Aαa	0.095 ± 0.007 Aαa	0.116 ± 0.006 Aαa
	S2	0.075 ± 0.006 Bαab	0.054 ± 0.007 Bαb	0.082 ± 0.006 Aαa	0.059 ± 0.006 Bαb
	S3	0.054 ± 0.007 Cαa	0.043 ± 0.007 Bαab	0.040 ± 0.006 Bαab	0.026 ± 0.004 Cαb
V2	S1	0.098 ± 0.006 Aαa	0.094 ± 0.004 Aαa	0.108 ± 0.004 Aαa	0.099 ± 0.005 Aβa
	S2	0.058 ± 0.007 Bβab	0.069 ± 0.005 Bαa	0.060 ± 0.005 Bβab	0.046 ± 0.005 Bαb
	S3	0.043 ± 0.008 Bαa	0.050 ± 0.007 Bαa	0.041 ± 0.008 Bαa	0.0286 ± 0.003 Bαa

Means followed by the same uppercase letter in the column do not differ significantly for salinity according to Tukey’s test (*p* ≤ 0.05). Means followed by the same Greek letter in the column do not differ significantly for variety according to Student’s *t*-test (*p* ≤ 0.05). Means followed by the same lowercase letter in the row do not differ significantly for Mg dose according to Tukey’s test (*p* ≤ 0.05). Standard error, n = 5.

**Table 8:** Transpiration (*E*, mmol H_2_O m^−2^ s^−1^) and leaf temperature (Tl, °C) in cowpea under different irrigation water salinity levels (S1—0.54 dS m^−1^; S2—3.50 dS m^−1^; S3—5.00 dS m^−1^).

Salinity	E	Tl
S1	2.95 ± 0.08 A	25.59 ± 0.04 C
S2	2.51 ± 0.08 B	26.21 ± 0.12 B
S3	2.33 ± 0.08 B	26.46 ± 0.10 A

Means followed by the same uppercase letter in the column do not differ significantly according to Tukey’s test (*p* ≤ 0.05). Standard error, n = 40.

**Table 9:** Stomatal conductance (*gs*, mol H_2_O m^−2^ s^−1^), internal CO_2_ concentration (*Ci*, µmol mol^−1^), and instantaneous water use efficiency (WUE*i*, [(µmol m^−2^ s^−1^)(mmol H_2_O m^−2^ s^−1^)^−1^]) in cowpea as affected by the interaction between irrigation water salinity levels (S1—0.54 dS m^−1^; S2—3.50 dS m^−1^; S3—5.00 dS m^−1^) and foliar Mg doses (0, 1, 2, and 3 mL L^−1^).

Salinity	Mg Doses (mL L^−1^)			
	*gs*			
	0	1	2	3
S1	0.161 ± 0.006 Aa	0.154 ± 0.011 Aa	0.146 ± 0.011 Aa	0.159 ± 0.009 Aa
S2	0.132 ± 0.010 Ba	0.128 ± 0.008 Ba	0.137 ± 0.005 ABa	0.121 ± 0.005 Ba
S3	0.130 ± 0.010 Ba	0.124 ± 0.007 Bab	0.124 ± 0.009 Bab	0.102 ± 0.003 Bb
	*Ci*			
S1	186.85 ± 5.19 Ca	181.75 ± 8.09 Ca	179.77 ± 5.99 Ca	179.26 ± 6.31 Ca
S2	218.29 ± 6.40 Ba	215.05 ± 6.51 Ba	209.12 ± 6.93 Ba	225.26 ± 6.50 Ba
S3	240.74 ± 6.43 Ab	240.66 ± 4.80 Ab	246.84 ± 7.62 Ab	273.53 ± 8.25 Aa
	*WUEi*			
S1	6.34 ± 0.19 Aa	6.26 ± 0.27 Aa	6.35 ± 0.23 Aa	6.56 ± 0.25 Aa
S2	5.18 ± 0.28 Ba	5.29 ± 0.25 Ba	5.59 ± 0.34 Aa	4.99 ± 0.26 Ba
S3	4.53 ± 0.19 Ba	4.69 ± 0.16 Ba	4.06 ± 0.23 Bab	3.47 ± 0.25 Cb

Means followed by the same uppercase letter in the column do not differ significantly for salinity (Tukey’s test, *p* ≤ 0.05). Means followed by the same lowercase letter in the row do not differ significantly for Mg dose (Tukey’s test, *p* ≤ 0.05). Standard error, n = 10.

**Table 10:** Internal CO_2_ concentration (*Ci*, µmol mol^−1^) in cowpea varieties (V1—‘Pingo de Ouro’; V2—‘Costela de Vaca’).

Variety	*Ci*
V1	212.35 ± 4.78 α
V2	220.51 ± 4.39 β

Means followed by the same Greek letter in the column do not differ significantly according to Student’s *t*-test (*p* ≤ 0.05). Standard error, n = 60.

**Table 11:** Chlorophyll *a* (Chl *a*), chlorophyll *b* (Chl *b*), and total chlorophyll (Chl *t*), expressed in ICF units, in cowpea varieties (V1—‘Pingo de Ouro’; V2—‘Costela de Vaca’) under different irrigation water salinity levels (S1—0.54 dS m^−1^; S2—3.50 dS m^−1^; S3—5.00 dS m^−1^).

Salinity	Chl *a*		Chl *b*		Chl *t*	
	Varieties					
	V1	V2	V1	V2	V1	V2
S1	31.31 ± 0.31 Aα	31.11 ± 0.38 Bα	20.35 ± 0.69 Aα	19.38 ± 0.77 Cα	51.76 ± 0.93 Bα	50.52 ± 1.09 Cα
S2	31.94 ± 0.29 Aα	31.99 ± 0.27 ABα	21.86 ± 0.68 Aα	22.24 ± 0.74 Bα	55.13 ± 1.12 Aα	55.03 ± 1.05 Bα
S3	31.50 ± 0.41 Aβ	32.71 ± 0.29 Aα	22.39 ± 0.84 Aβ	25.44 ± 0.71 Aα	54.35 ± 1.11 Aβ	58.94 ± 0.75 Aα

Means followed by the same uppercase letter in the column do not differ significantly for salinity (Tukey’s test, *p* ≤ 0.05). Means followed by the same Greek letter in the row do not differ significantly for variety (Student’s *t*-test, *p* ≤ 0.05). Standard error, n = 20.

**Table 12:** Leaf magnesium content (Mg, g kg^−1^) in cowpea under different irrigation water salinity levels (S1—0.54 dS m^−1^; S2—3.50 dS m^−1^; S3—5.00 dS m^−1^).

Salinity	Mg
S1	6.231 ± 0.266 A
S2	5.026 ± 0.261 B
S3	4.486 ± 0.266 B

Means followed by the same uppercase letter in the column do not differ significantly according to Tukey’s test (*p* ≤ 0.05). Standard error, n = 40.

**Table 13:** Chemical characterization of the irrigation water sources.

Water	pH	EC	K^+^	Na^+^	Ca^2+^	Mg^2+^	Cl^-^	CO_3_^2−^	HCO_3_^−^	SAR	Hardness
Sources		dS m^−1^	---------------------------- mmol_c_ L^−1^ ------------------------------								mg L^−1^
Saline reject	7.28	7.40	0.42	27.98	20.30	17.05	67.00	0.07	1.72	6.47	1867.50
S1	7.65	0.54	0.22	3.72	0.63	0.41	2.40	0.68	3.11	5.18	51.75
S2	7.68	3.50	0.30	13.68	8.00	6.90	26.00	0.19	1.28	5.01	745.00
S3	7.70	5.00	0.35	18.30	13.15	9.00	41.00	0.10	2.14	5.50	1107.50

Saline reject = from the desalination unit at the Jurema settlement, Mossoró-RN; S1 = local supply water; S2 = mixture of 55% supply water + 45% brine; S3 = mixture of 33% supply water + 67% brine. According to the USSL irrigation water classification diagram (Richards, 1954), the water sources used in this experiment can be classified as follows: Brine = C5S3; S_1_ = C2S1; S_2_ = C4S2; S3 = C5S2. EC = Electrical conductivity. RAS = Sodium adsorption rate obtained by the equation (SAR = Na^+^/(Ca^2+^ + Mg^2+^)^0.5^/2).

**Table 14:** Chemical and physical characterization of the soil prior to cowpea cultivation.

Chemical Characterization																				
N	O.M.	P			K^+^		Na^+^	Ca^2+^		Mg^2+^	Al^3+^		H + Al	SB	t	CEC		V	m	PES
g kg^−1^		----- mg dm^−3^ ----						------------------- cmol_c_ dm^−3^-----------------										------ % --------		
0.47	14.00	90.80			61.80		48.60	2.44		0.68	0		0	3.50	3.50	3.50		100.00	0	6.00
**Physical characterization**																				
Ds	Sand Clay Silt																	Texture class		
kg dm^−3^	-------------------------- g kg^−1^ -------------------------------																	Sandy loam		
1.6	820 30 150																			
**Soil saturation extract**																				
**pH**	**EC**		**K^+^**	**Na^+^**		**Ca^2+^**			**Mg^2+^**			Cl^−^			CO_3_^2−^		HCO_3_^−^			
	**dS m^−1^**		**------------------------------------------------ mmol_c_ L^−1^ ---------------------------------------------**																	
7.30	0.83		0.57	4.05		2.95			4.55			5.0			0		9.1			

Soil pH was measured in a 1:2.5 soil-to-water ratio. Electrical conductivity (EC) was determined in the 1:2.5 soil-to-water extract. The nutrients P, Na^+^, and K^+^ were extracted using Mehlich-1 in a 1:10 soil-to-extractant ratio.

**Table 15:** Chemical composition of the foliar fertilizer Liqui-Plex Fruit^®^.

Nutrient Composition								
N	Ca	S	B	Cu	Mn	Mo	Zn	O.C.
--------------------------------------------------- g L^−1^ ----------------------------------------------								%
73.50	14.70	77.91	14.70	0.74	73.50	1.47	73.50	2.35

N—Nitrogen; Ca—Calcium; S—Sulfur; B—Boron; Cu—Copper; Mn—Manganese; Mo—Molybdenum; Zn—Zinc; O.C.—organic carbon.

**Table 16:** Salt volume applied to the soil through each salinity level of the irrigation water.

EC_w_	Irrigation Volume	Salts Added via Irrigation Water
	L per Pot	g per Pot
S1	46.388	14.844
S2	30.221	67.695
S3	23.195	74.224

**Table 17:** Soil electrical conductivity at the beginning of the experiment and at 71 DAS, as affected by irrigation water salinity.

Salinity	Soil EC (dS m^−1^)	
	1 DAS	71 DAS
S1	0.83	2.07
S2	0.83	9.32
S3	0.83	15.38

## Data Availability

Data is contain in the main text.

## References

[1] Melo A.S., Melo Y.L., Lacerda C.F., Viégas P.R.A., Ferraz R.L.S., Gheyi H.R. (2022). Water restriction in cowpea plants [*Vigna unguiculata* (L.) Walp.]: Metabolic changes and tolerance induction. Rev. Bras. Eng. Agrícola Ambient..

[2] Sousa G.A., Siviero A., Braga A.S., Bassinello P.Z., Santos R.C., Felisberto F.Á.V., Teixeira M.C. (2023). Qualidade nutricional e armazenamento de variedades de feijão-caupi cultivados no Juruá, Acre (Portuguese). Desarro. Local Sosten..

[3] FAO—Food and Agriculture Organization (2018). FAOSTAT. Crops. Cowpeas, Dry. http://faostat.fao.org/browse/Q/QC/E.

[4] CONAB—Companhia Nacional de Abastecimento (2024). Boletim da safra de Grãos. 8° Levantamento, Safra 2023/24, Edição Grãos (Portuguese). https://www.conab.gov.br/info-agro/safras/graos/boletim-da-safra-de-graos/.

[5] CONAB—Companhia Nacional de Abastecimento (2021). Perspectivas para a Agropecuária. https://www.conab.gov.br/perspectivaspara-a-agropecuaria.

[6] Sousa T.J.F., Rocha M.M., Damasceno-Silva K.J., Bertini C.H.C.M., Silveira L.M., Sousa R.R., Sousa J.L.M. (2019). Simultaneous selection for yield, adaptability, and genotypic stability in immature cowpea using REML/BLUP. Pesqui. Agrop. Bras..

[7] Rolim R.R., Nascimento N.F.F., Nascimento M.F., Araujo H.F.P. (2023). Genotype × environment interaction and stability in landraces of cowpea under dryland conditions. Rev. Caatinga.

[8] Rivas R., Falcão H.M., Ribeiro R.V., Machado E.C., Pimentel C., Santos M.G. (2016). Drought tolerance in cowpea species is driven by less sensitivity of leaf gas exchange to water deficit and rapid recovery of photosynthesis after rehydration. S. Afr. J. Bot..

[9] Melo A.S., Silva A.R.F., Dutra A.F., Dutra W.F., Brito M.E.B., Sá F.V.d.S. (2018). Photosynthetic efficiency and production of cowpea cultivars under deficit irrigation. Rev. Amb. Água.

[10] Bezerra R.U., Viana T.V.A., Azevedo B.M., Pereira Filho J.V., Lima A.D. (2020). Production and quality of Maranhão pumpkin under influence of water depths and nitrogen doses. Irriga.

[11] Gomes B.P., Gomes F.I.B.P., Zanella M.E. (2023). History, cause and particulars of climate semiarid of Northeast of Brazil (Spanish). Geografares.

[12] Dias N.S., Silva J.F., Moreno-Pizani M.A., Lima M.C.F., Ferreira J.F.S., Linhares E.L.R., Sousa Neto O.N., Portela J.C., Silva M.R.F., Ferreira Neto M., Taleisnik E., Lavado R.S. (2021). Environmental, agricultural, and socioeconomic impacts of salinization to family-based irrigated agriculture in the brazilian semiarid region. Saline and Alkaline Soils in Latin America.

[13] Loiola A.T., Sá F.V.d.S., Ferreira Neto M., Torres S.B., Praxedes S.S.C., Reges L.B.L., Oliveira R.R., Alves T.R.C. (2022). Phenology and production of traditional seeds of cowpea irrigated with saline water. Rev. Ciênc. Agron..

[14] Ayers R.S., Westcot D.W. (1985). Water Quality for Agriculture.

[15] Sá F.V.d.S., Ferreira-Neto M., Lima Y.B., Paiva E.P.d., Prata R.C., Lacerda C.F.F., Brito M.E.B. (2018). Growth, gas exchange and photochemical efficiency of the cowpea bean under salt stress and phosphorus fertilization. Comun. Sci..

[16] Nascimento E.C.S., Souza A.R., Nascimento R., Silva A.A.R., Bezerra C.V.C., Lima R.F., Guimarães R.F.B., Batista M.C. (2023). Co-inoculation with *Bradyrhizobium* spp. and *Azospirillum brasilense* in cowpea under salt stress. Rev. Bras. Eng. Agríc. Ambient..

[17] Sá F.V.d.S., Silva I.E., Ferreira-Neto M., Lima Y.B., Paiva E.P., Gheyi H.R. (2021). Phosphorus doses alter the ionic homeostasis of cowpea irrigated with saline water. Rev. Bras. Eng. Agrícola Ambient..

[18] Ayalew T., Yoseph T., Hoegy P., Cadisch G. (2021). Yield response of field-grown cowpea varieties to *Bradyrhizobium* inoculation. Agron. J..

[19] Lima M.A., Castro V.F., Vidal J.B., Enéas-Filho J. (2011). Silicon application on plants of maize and cowpea under salt stress. Rev. Ciênc. Agron..

[20] Silva A.A.R., Lima G.S., Azevedo C.A.V., Veloso L.L.S.A., Souza L.P., Fátima R.T., Silva F.A., Gheyi H.R. (2023). Exogenous application of salicylic acid on the mitigation of salt stress in *Capsicum annuum* L. *Ciênc*. Rural.

[21] Silva A.A.R., Lima G.S., Azevedo C.A.V., Arruda T.F.L., Gheyi H.R., Soares L.A.A. (2023). Salicylic acid attenuates the harmful effects of salt stress on the morphophysiology of early dwarf cashew. Ciênc. E Agrotec..

[22] Jales Filho R.C., Melo Y.L., Viégas P.R.A., Oliveira A.P.S., Almeida Neto V.E., Ferraz R.L.S., Gheyi H.R., Carol P., Lacerda C.F., Melo A.S. (2023). Salicylic acid and proline modulate water stress tolerance in a traditional variety of cowpeas. Rev. Bras. Eng. Agrícola Ambient..

[23] Rodrigues Filho R.A., Nobre R.G., Lima G.S.d., Moraes F.M.d.S., Soares L.A.d.A., Teixeira A.D.d.S., Peixoto T.D.C., Vasconcelos E.d.S. (2023). Production of guava seedlings with increasing water salinity and nitrogen-potassium fertilizations. Rev. Caatinga.

[24] Cakmak I., Hengeler C., Marschner H. (1994). Partitioning of shoot and root dry matter and carbohydrates in bean plants suffering from phosphorus, potassium and magnesium 76 deficiency. J. Exp. Bot..

[25] Hermans C., Johnson G.N., Strasser R.J., Verbruggen N. (2004). Physiological characterization of magnesium deficiency in sugar beet: Acclimation to low magnesium differentially affects photosystems I and II. Planta.

[26] Geiger D. (2011). Plant sucrose transporters from a biophysical point of view. Mol. Plant.

[27] Tränknera M., Tavakolb E., Jákli B. (2018). Functioning of potassium and magnesium in photosynthesis, photosynthate translocation and photoprotection. Physiol. Plant..

[28] Atta K., Mondal S., Gorai S., Singh A.P., Kumari A., Ghosh T., Roy A., Hembram S., Gaikwad D.J., Mondal S. (2023). Impacts of salinity stress on crop plants: Improving salt tolerance through genetic and molecular dissection. Front. Plant Sci..

[29] Ahmed N., Zhang B., Bozdar B., Chachar S., Rai M., Li J., Li Y., Hayat F., Chachar Z., Tu P. (2023). The power of magnesium: Unlocking the potential for increased yield, quality, and stress tolerance of horticultural crops. Front. Plant Sci..

[30] Praxedes S.S.C., Ferreira Neto M., Loiola A.T., Santos F.J.Q., Umbelino B.F., Silva L.A., Moreira R.C.L., Melo A.S., Lacerda C.F., Fernandes P.D. (2022). Photosynthetic responses, growth, production, and tolerance of traditional varieties of cowpea under salt stress. Plants.

[31] Novais R.F., Neves J.C.L., Barros N.F., Oliveira A.J. (1991). Ensaio em ambiente controlado. Métodos de Pesquisa em Fertilidade do Solo.

[32] Marschner P. (2012). Marschner’s Mineral Nutrition of Higher Plants.

[33] Andrade J.R., Maia Júnior S., Barbosa J.W.S., Alencar A.E.V., Jovino R.S., Nascimento R. (2019). Chlorophyll fluorescence as a tool to select salinity-tolerant cowpea genotypes. Comun. Sci..

[34] Araújo E.D., Melo A.S., Rocha M.S., Carneiro R.F., Rocha M.M. (2017). Genotypic variation on the antioxidative response of cowpea cultivars exposed to osmotic stress. Rev. Caatinga.

[35] Oliveira L.K.B., Costa R.S., Silva J.S., Silva B.A., Lima K.V.G., Pinto M.B.S., Batista A.B.P., Silva F.J.L., Silva T.I., Mesquita R.O. (2024). Morphophysiology of cowpea under salt stress and application of carbon-based nanobiostimulant in the vegetative stage. Rev. Bras. Eng. Agrícola Ambient..

[36] Santos A.R., Melo Y.L., Oliveira L.F., Cavalcante I.E., Ferraz R.L.S., Sá F.V.S., Lacerda C.F.F., Melo A.S. (2022). Exogenous silicon and proline modulate osmoprotection and antioxidant activity in cowpea under drought stress. J. Soil Sci. Plant Nutr..

[37] Guo W., Nazim H., Liang Z., Yang D. (2016). Magnesium deficiency in plants: An urgent problem. Crop J..

[38] Munns R., Tester M. (2008). Mechanisms of Salinity Tolerance. Annu. Rev. Plant Biol..

[39] Li J., Yokosho K., Liu S., Cao H.R., Yamaji N., Zhu X.G., Liao H., Ma J.F., Chen Z.C. (2020). Diel magnesium fluctuations in chloroplasts contribute to photosynthesis in rice. Nat. Plants.

[40] Tian X.Y., He D.D., Bai S., Zeng W.Z., Wang Z., Wang M., Wu L.Q., Chen Z.C. (2021). Physiological and molecular advances in magnesium nutrition of plants. Plant Soil.

[41] Inoue S., Hayashi M., Huang S., Yokosho K., Gotoh E., Ikematsu S., Okumura M., Suzuki T., Kamura T., Kinoshita T. (2022). A tonoplast-localized magnesium transporter is crucial forstomatal opening in Arabidopsis under high Mg^2+^ conditions. New Phytol..

[42] Garcia A., Crusciol C.A.C., Rosolem C.A., Bossolani J.W., Nascimento C.A.C., Mccray J.M., Reis A.R., Cakmak I. (2022). Potassium-magnesium imbalance causes detrimental effects on growth, starch allocation and rubisco activity in sugarcane plants. Plant Soil.

[43] Wakeel A., Ishfaq M. (2022). Phytoavailability of potassium. Potash Use and Dynamics in Agriculture.

[44] Mukherjee S., Mukherjee A., Das P., Bandyopadhyay S., Chattopadhyay D., Chatterjee J., Majumder A.L. (2021). A salt-tolerant chloroplastic FBPase from Oryza coarctata confers improved photosynthesis with higher yield and multi-stress tolerance to indica rice. Plant Cell.

[45] Cakmak I., Kirkby E.A. (2008). Role of magnesium in carbon partitioning and alleviating photooxidative damage. Physiol. Plant..

[46] Rengel Z., Bose J., Chen Q., Tripathi B.N. (2015). Magnesium alleviates plant toxicity of aluminium and heavy metals. Crop Pasture Sci..

[47] Jiao J., Li J., Chang J., Li J., Chen X., Li Z., Song Z., Xie D., Zhang B. (2023). Magnesium Effects on Carbohydrate Characters in Leaves, Phloem Sap and Mesocarp in Wax Gourd (*Benincasa hispida* (Thunb.) Cogn.). Agronomy.

[48] Li J., Muneer M.A., Sun A., Guo Q., Wang Y., Huang Z., Li W., Zheng C. (2023). Magnesium application improves the morphology, nutrients uptake, photosynthetic traits, and quality of tobacco (*Nicotiana tabacum* L.) under cold stress. Front. Plant Sci..

[49] Alvares C.A., Stape J.L., Sentelhas P.C., Gonçalves J.L.d.M., Sparovek G. (2013). Köppen’s climate classification map for Brazil. Meteorol. Z..

[50] Almeida I.C.F. (2016). Eficiência do uso de Fósforo em Genótipos de Feijão Caupi (Portuguese). Master’s Thesis.

[51] Medeiros J.F. (1992). Qualidade da Água de Irrigação e Evolução da Salinidade nas Propriedades Assistidas Pelo “GAT” nos Estados do RN, PB e CE (Portuguese). Thesis, Universidade Federal da Paraíba, Campina Grande, Brazil. http://dspace.sti.ufcg.edu.br:8080/jspui/handle/riufcg/2896.

[52] Rhoades J.D., Kandiah A., Mashali Q.M. (1992). The Use of Saline Waters for Crop Production.

[53] Santos H.G., Jacomine P.K.T., Anjos L.H.C., Oliveira V.A., Lumbreras J.F., Coelho M.R., Almeida J.A., Araújo Filho J.C., Oliveira J.B., Cunha T.J.F. (2018). Sistema Brasileiro de Classificação de Solos.

[54] USDA—United States Department of Agriculture, Soil Survey Staff (2014). Keys to Soil Taxonomy.

[55] Richards L.A. (1954). Diagnosis and Improvement of Saline and Alkali Soils.

[56] Teixeira P.C., Donagemma G.K., Fontana A., Teixeira W.G. (2017). Manual de Métodos de Análises de Solo.

[57] Bernardo S., Soares A.A., Mantovani E.C. (2006). Manual de Irrigação.

[58] Fernandes C.S., Sá F.V.d.S., Ferreira Neto M., Dias N.d.S., Reges L.B.L., Gheyi H.R., Paiva E.P., Silva A.A., Melo A.S. (2022). Ionic homeostasis, biochemical componentes and yield of italian zucchini under nitrogen forms and salt stress. Braz. J. Biol..

[59] EMBRAPA. Empresa Brasileira de Pesquisa Agropecuária (2009). Manual de Análises Químicas de Solos, Plantas e Fertilizantes.

[60] Ferreira D.F. (2019). Sisvar: A computer analysis system to fixed effects split plot type designs. Braz. J. Biom..

